# ﻿Back to the roots: Uncovering ectomycorrhizal communities across three major African vegetation types

**DOI:** 10.3897/imafungus.16.147055

**Published:** 2025-05-29

**Authors:** Lowie Tondeleir, Eske De Crop, Tatiana Semenova, Jorinde Nuytinck, André-Ledoux Njouonkou, Atsu Kudzo Guelly, Glen Dierickx, József Geml, Annemieke Verbeken

**Affiliations:** 1 Ghent University, Ghent, Belgium Ghent University Ghent Belgium; 2 Meise Botanic Garden, Brussels, Belgium Meise Botanic Garden Brussels Belgium; 3 Naturalis Biodiversity Center, Leiden, Netherlands Naturalis Biodiversity Center Leiden Netherlands; 4 University of Bamenda, Bambili, Cameroon University of Bamenda Bambili Cameroon; 5 Université de Lomé, Lomé, Togo Université de Lomé Lomé Togo; 6 Research Institute for Nature and Forest, Geraardsbergen, Belgium Research Institute for Nature and Forest Geraardsbergen Belgium; 7 Eszterházy Károly Catholic University, Eger, Hungary Eszterházy Károly Catholic University Eger Hungary

**Keywords:** eDNA, fungal community, Miombo woodland, rainforest, *Russulaceae*, Species Hypothesis, Sudanian woodland

## Abstract

Ectomycorrhizal fungi (EcM) are critical to the health and sustainability of many African ecosystems that include EcM-associated tree species. In Sub-Saharan Africa, three major EcM-dominated vegetation types can be distinguished: the Central African Guineo-Congolian rainforests, the West African Sudanian woodlands and the East African Zambezian Miombo woodlands. While the rainforests feature humid conditions with isolated patches of EcM trees amongst predominantly arbuscular mycorrhizal (AM) communities, the woodlands are characterised by drier soils and more vast continuous areas of EcM trees. We hypothesise that the isolation of EcM tree patches within the rainforest promotes a unique and potentially endemic EcM fungal community, while riparian forests found along rivers in woodland areas may serve as corridors, facilitating the spread of such rare taxa into woodland regions.

In this study, we employ root tip metabarcoding combined with Species Hypothesis (SH) matching to characterise the EcM communities across these three vegetation types. Consistent with previous findings from fruit-body surveys and eDNA studies, our results show that *Russulaceae* is the most abundant EcM clade across all three regions. Other clades reveal greater discrepancy compared to their above-ground abundances, with notably high abundances of *Inocybaceae*, *Thelephoraceae* and *Sebacinaceae*, especially in woodlands. Conversely, *Amanitaceae* and *Boletaceae* appear under-represented. Both *Boletaceae* and *Elaphomycetaceae* are found to be more prevalent in rainforest and riparian zones, illustrating the unique EcM community of the Guineo-Congolian rainforest. Our findings highlight the corridor potential of riparian areas in facilitating the spread of these rainforest endemics. This suggests that local edaphic and climatic conditions can override broad spatial patterns, such as distance decay, in community structure of African EcM. Moreover, we suggest a stronger effect of EcM host specificity than previously suggested for African fungal communities.

Lastly, we assess the level of species-level representation and accuracy of taxonomic annotation of SHs within African *Lactifluus*. We confirm it to be one of the most thoroughly described and collected fungal genera on the continent, with over 80% of identified SHs represented in our herbarium collections.

## ﻿Introduction

### ﻿Three main ectomycorrhizal vegetation types in sub-Saharan Africa

Ectomycorrhizal (EcM) fungi perform a major ecological role in nearly all terrestrial ecosystems world-wide. They establish mutualistic associations with many plant species, enhancing nutrient and water uptake, thereby contributing to the overall productivity and biodiversity of these ecosystems. In many regions, researchers have studied the composition and distribution of EcM communities (Peay et al. 2008; Tedersoo et al. 2009, 2014b). However, one of the largest forested regions dominated by EcM trees, sub-Saharan Africa, has been largely overlooked. Sub-Saharan Africa can be roughly characterised by three vegetation types in which ectomycorrhizal associations are prominent: Central-African Guineo-Congolian rainforests in the Congo Basin, West-African Sudanian woodlands and East-African Zambezian Miombo woodlands (White 1983).

In the Guineo-Congolian Region of Central Africa, rainforests represent the dominant vegetation type (White 1983). These forests are characterised by consistently high humidity, driven by high annual rainfall during distinct rainy seasons. Their soils are typically acidic and nutrient-poor, particularly deficient in phosphorus, posing challenges for tree growth and, thus, shaping the importance of mycorrhizal symbioses. Common EcM-associated trees in these forests include species of the genus Uapaca (Phyllanthaceae) and *Gilbertiodendrondewevrei* (*Fabaceae*, subfamily *Detarioideae*, tribe *Amherstieae*) (Sonké and Couvreur 2014). *Uapaca* species generally grow intermixed with other tree species, while *G.dewevrei* forms monodominant stands (Michaëlla Ebenye et al. 2017; Hall et al. 2020). These EcM-dominated areas are patchily distributed within larger arbuscular mycorrhiza (AM)-dominated stands. In mixed *Uapaca* stands, the hypothesised low host-specificity of EcM fungi provides a competitive advantage over the predominantly AM-associated canopy species (Diédhiou et al. 2010). This patchy distribution of EcM trees has also been linked to a high degree of fungal endemism (Heimpel et al. 2024).

The Sudanian woodlands of West Africa consist of open woodlands, tree savannahs and grasslands that grow on dry, phosphorus – and nitrogen-depleted soils with ample sunlight. These woodlands experience a pronounced dry season lasting several months, with seasonal rains concentrated in a brief period. Important EcM host trees in this region include species of *Uapaca*, *Isoberlinia* (*Fabaceae*, subfamily *Detarioideae*, tribe *Amherstieae*) and Monotes (Dipterocarpaceae) (Houdanon et al. 2019). These trees are known to form symbiotic relationships with EcM fungi as an adaptation to the dry and nutrient-poor conditions (Yorou et al. 2014).

Miombo woodland, the most extensive vegetation type in Africa that is dominated by EcM associations, spans approximately 3.6 million km² across east, central and southern Africa (White 1983; Frost 1996; Timberlake and Chidumayo 2011). These seasonally dry, deciduous woodlands are characterised by a short rainy season and a prolonged dry season, with frequent fires as a result. The term “Miombo” derives from the vernacular names for trees in the *Brachystegiaboehmii* – *longifolia* group in several African languages and reflects the dominance of leguminous trees of inter alia the genera *Brachystegia* (*Fabaceae*, subfamily *Detarioideae*, tribe *Amherstieae*), *Julbernardia* (*Fabaceae*, subfamily *Detarioideae*, tribe *Amherstieae*) and *Isoberlinia*, all of which form associations with EcM fungi (Smith and Allen 2004). This dominance of EcM trees is a response to the region’s nutrient-poor soils, which vary in mineral composition, but are typically low in organic matter and nitrogen due to recurring fires (Frost 1996). Thus, EcM fungi play an essential role in enhancing tree fitness by improving nitrogen uptake, enabling these trees to thrive in these porous, infertile soils (Högberg and Nylund 1981; Högberg and Alexander 1995).

### ﻿Riparian forests as corridors

Riparian forests, also known as gallery forests, are specialised ecosystems found along rivers and streams within various African landscapes. These forests form narrow bands of dense vegetation that cut through drier ecosystems, such as savannahs and woodlands. Due to their linear shape, riparian forests hold significant potential as ecological corridors. Despite their importance as habitats for threatened wildlife and vegetation, they remain understudied in many African regions (Naiman and Decamps 1997; Gautier-Hion and Brugière 2005). The conservation value of these forest corridors lies in their ability to create habitat linkages and provide dispersal opportunities for wildlife in human-dominated landscapes.

This corridor potential has also been hypothesised to benefit EcM fungi, given the fact that EcM-forming *Uapaca* species often dominate these forested patches (Mony et al. 2022). Although riparian areas in rainforests and woodlands may be separated by vast geographic distances, field observations suggest that their fungal communities share similarities. Riparian zones appear to host overlapping fungal communities from both rainforests and woodlands (Meidl et al. 2021). By offering similar humid and acidic soil conditions, riparian forests may act as a corridor and a refuge for the unique and possibly endemic fungal EcM communities associated with rainforests.

### ﻿Below – and above-ground EcM diversity in Africa

The diversity of EcM trees across African vegetation types supports a vast array of fungal species, many of which form prominent fruit-bodies. Consequently, most data on these EcM fungal communities have been derived from fruit-body surveys. Many EcM fungi produce edible basidiocarps, making them important non-timber forest products for rural communities. Thus, surveys in these areas often focus on edible species due to their local economic and nutritional value (Ndong et al. 2011; Milenge Kamalebo et al. 2018; De Kesel et al. 2024). However, EcM fungal diversity in African rainforests is particularly understudied compared to woodlands, as rainforest fungi are less frequently harvested for food (Milenge Kamalebo et al. 2018; Milenge Kamalebo and De Kesel 2020). As a result, existing studies are often geographically and taxonomically limited.

Based on above-ground biodiversity records, the *Russulaceae* family, especially the genera *Russula* and *Lactifluus*, dominates tropical African ecosystems in both species richness and abundance (Verbeken and Buyck 2002; Corrales et al. 2022). Other prominent groups include the *Boletaceae*, *Hydnaceae* (formerly *Cantharellaceae*) and *Amanitaceae* (Verbeken and Buyck 2002; Bâ et al. 2012; Tedersoo and Smith 2013, 2017; Corrales et al. 2022). In contrast to temperate regions, the families *Cortinariaceae* and, to a lesser extent, *Inocybaceae* are under-represented in tropical Africa. Interestingly, early soil diversity studies revealed that *Inocybaceae* and *Thelephoraceae* rank second and third to *Russulaceae* in below-ground species richness (Bâ et al. 2012; Meidl et al. 2021). The high below-ground abundance of *Thelephoraceae*, a group with few conspicuous fruit-body-forming species, underscores the limitations of fruit-body surveys in fully capturing EcM fungal diversity (Taylor et al. 2000; Horton and Bruns 2001).

Given the limited exploration of major African vegetation types using molecular approaches, such as environmental DNA (eDNA) metabarcoding, this study investigates below-ground EcM biodiversity in the Guineo-Congolian Region, Miombo and Sudanian woodlands. Riparian forests within these vegetation types are examined as subtypes. By analysing root-tip samples through metabarcoding and Species-Hypothesis (SH) matching, we aim to evaluate the following hypotheses:

Each of the three forest types (Guineo-Congolian Region, Sudanian woodland and Miombo woodland) hosts a unique EcM community composition, with greater shared species diversity between the two woodland types than between rainforests and woodlands. The distinct and unique EcM diversity of rainforests is partially shared with riparian forests in all three regions, enforcing their role as ecological corridors.

Secondly, we want to estimate how well extensive fruit-body collections cover the below-ground diversity as captured by root tip metabarcoding and evaluate the accuracy of the taxonomic annotation and species delimitation of the applied SH-matching tool. To do so, we compare *Lactifluus* Species Hypothesis annotations from this dataset with specimens in the GENT fungarium collection. The genus Lactifluus (Russulaceae) is particularly well-studied in tropical Africa due to extensive phylogenetic and taxonomic research over the past decades (De Crop et al. 2021). Moreover, the long-standing tradition of studying (African) *Russulaceae* at Research Group Mycology, Ghent University, has resulted in an extensive collection of *Lactifluus* at the GENT fungarium (Verbeken 1995, 1996, 1998; Verbeken and Walleyn 1999; Verbeken et al. 2000, 2012; De Crop et al. 2012, 2016, 2017, 2019; Delgat et al. 2017; De Lange et al. 2018).

## ﻿Methods

### ﻿Study areas and sampling

**Figure 1.**
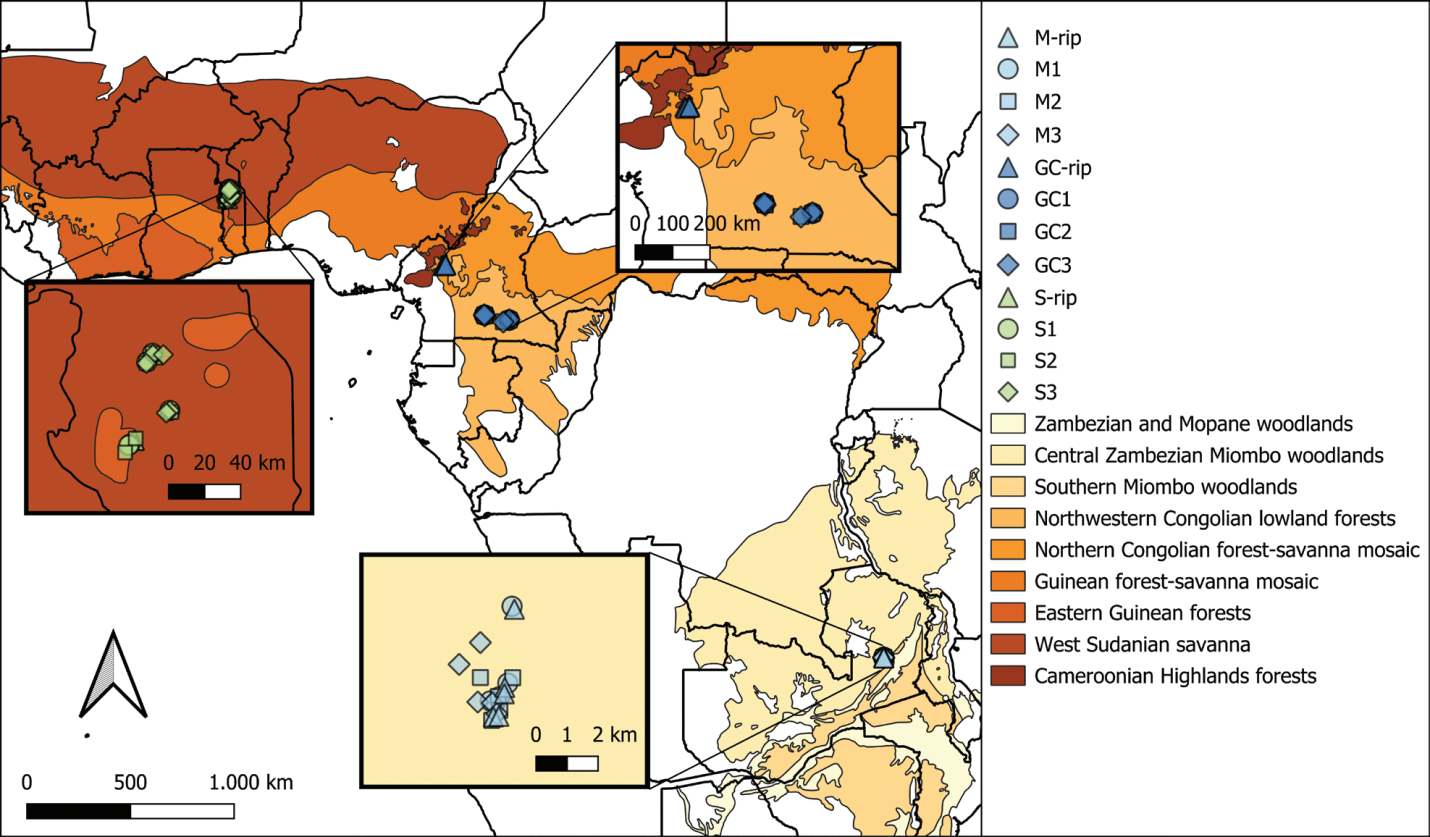
Map of sampling locations across Africa. Sudanian woodland (S) samples were collected in Togo (West-Sudanian savannah), Guineo-Congolian (GC) in Cameroon (north-western Congolian lowland forests & northern Congolian forest-savannah mosaic) and Miombo woodland (M) in Zambia (central Zambezian Miombo woodlands). Shapes indicate forest subtype within vegetation types, with ‘rip’ as riparian forests, with main vegetation types annotated according to Olson et al. (2001).

We sampled the three main EcM vegetation types in sub-Saharan Africa in three different countries: Zambezian Miombo woodland in Zambia, Guineo-Congolian rainforest in Cameroon and Sudanian woodland in Togo. For each of these vegetation types, four subtypes were selected in the field. Within each subtype, five plots were chosen (Fig. 1).

Samples from within the Zambezian Miombo woodlands were collected in the woodlands of Mutinondo Wilderness lodge. This part of Miombo woodland is co-dominated by *Brachystegiaspiciformis*, *B.utilis*, *B.floribunda* and *Julbernadiapaniculata* (Smith and Allen 2004). The following subtypes were selected: (i) Miombo along the path (M1); (ii) dense Miombo (M2); (iii) open Miombo (M3); and (iv) Miombo riparian forest (M-rip), characterised by *Uapacalissopyrena* as the dominant canopy species. The Guineo-Congolian Region was sampled in two locations. Rainforest samples from the Guineo-Congolian Region were collected in Cameroon, in the Dja Biosphere Reserve and the riparian samples were taken in the gallery forests of the Noun division in the West region. The following subtypes were selected: (i) *Uapaca* non-flooded forest, with *U.guineensis* as the main EcM tree (GC1); (ii) *Uapaca* along the river, with *U.guineensis* as main EcM tree (GC2); (iii) monodominant *Gilbertiodendrondewevrei* forest (GC3); and (iv) Guineo-Congolian riparian forest, characterised by *U.guineensis* as the main EcM tree (GC-rip). The Sudanian woodlands were sampled in the Fazao-Malfakassa National Park in Togo. We distinguished the following subtypes: (i) woodland dominated by *Isoberliniadoka* (S1); (ii) woodland dominated by *U.togoensis* (S2); (iii) woodland dominated by *Monotes* spp. (S3); (iv) Sudanian riparian forest, dominated by *U.guineensis* (S-rip).

To gain the most representative image of the established (i.e. colonising the roots of the host tree) EcM community composition, root tips were isolated for sequencing instead of whole soil samples. At each plot, 40 soil cores (15 cm in depth and 5 cm in diameter) were collected after removing the litter layer, maintaining a minimum distance of two metres between samples and then pooled. Per plot, root tips were sorted out, cleaned with water and stored in CTAB buffer. A subsample of the homogenised soil was taken to determine soil characteristics. The concentration of nitrogen and carbon was determined through combustion at 1150°C, after which the gases were measured by a thermal conductivity detector in a CNS elemental analyser (Vario Macro Cube, Elementar, Germany). Total phosphorus was measured colourimetrically according to the malachite green procedure (Lajtha et al. 1999), after complete destruction with HClO_4_ (65%), HNO_3_ (70%) and H_2_SO_4_ (98%) in Teflon bombs for 4 h at 150°C. The percentage of organic material was determined after combustion of soil during 4 h, gradually increasing the temperature to 450°C. Lastly, pH-H_2_O was determined by shaking a 1:5 ratio soil/H_2_O mixture for 5 min at 300 rpm and measuring with a pH meter Orion 920A with pH electrode model Ross sure-flow 8172 BNWP (Thermo Scientific Orion, USA).

A total of 60 samples were collected: 20 per vegetation type, with five per subtype (1 sample failed amplification in the riparian plot of the Sudanian woodland). An overview of the metadata and soil characteristics of these samples can be found in Suppl. material 2.

### ﻿Molecular work

DNA extraction, PCR protocol, Ion Torrent sequencing and data clean-up procedures follow Geml et al. (2014). Briefly, for each sample, two independent DNA extractions were performed using NucleoSpin Soil and Plant kits (Macherey-Nagel Gmbh and Co., Düren, Germany), using ca. 1 ml of lyophilised root tips and pooled duplicates to optimise extraction homogenisation. Due to high concentration of pigments in extracted DNA, eluate was additionally purified using Promega DNA Clean-Up kit (Promega Benelux, Netherlands) prior to further processing. We used primers fITS7 and ITS4 to amplify the ITS2 rDNA region, using the following PCR settings: 95°C for 2 min., 25 cycles of 95°C for 30 sec., 54°C for 1 min., 72°C for 2 min. and 72°C for 10 min. (White et al. 1990; Ihrmark et al. 2012). The ITS4 primer was labelled with sample-specific Multiplex Identification DNA-tags (MIDs). The amplicon library was sequenced using an Ion 318TM Chip by an Ion Torrent Personal Genome Machine (PGM; Life Technologies, Guilford, CT, U.S.A.) at Naturalis Biodiversity Center, which produced 2,680,536 single-end reads with an average read length of 269 bp.

### ﻿Bioinformatic and statistical analyses

The ITS2 region was extracted from demultiplexed raw reads using ITSXPRESS (Einarsson and Rivers 2024). VSEARCH was used to remove putative chimeric sequences through both de-novo and reference-based filtering (UNITE, UCHIME release v.9.0) and to filter out reads with an expected error > 1 (Rognes et al. 2016; Abarenkov et al. 2024). Sequences were dereplicated and subjected to SH MATCHING at the 2% threshold (UNITE General FASTA release v.10.0) (Abarenkov et al. 2022, 2024). Dereplicated sequences identified to existing SHs or classified as new SHs in existing compound clusters were retained. The other reads, which were either identified as chimeric or formed new compound clusters, were discarded. The resulting SH matches were used to sum the conspecific abundances into an SH-table, which was used for the following analyses. Subsequent analyses were performed in R 4.2.3, using PHYLOSEQ 1.42.0 (McMurdie and Holmes 2013; R Core Team 2023). SHs with a total read abundance below 5 were discarded, as this proved to remove many new singleton SHs, which are largely unidentifiable to the family level. To evaluate the effect of sequencing depth, the data were rarefied to the lowest sample size. As rarefied data consistently showed similar patterns to non-transformed data, no rarefaction was performed in further analyses. Rather, we corrected for differences in sampling depth by including the logarithmic transformation of the sequencing depth as a covariate in relevant statistical tests. Lastly, the sequencing depth and sampling depth in terms of number of recovered SHs were visualized using iNEXT 3.0.1 (Chao et al. 2014) (Suppl. material 1: fig. S1).

SHs were assigned to their ecological guild using their genus-level classification, using the FungalTraits V1.2 database (Põlme et al. 2020). The SH-table was filtered for EcM and this subset was used for the analyses. Differences in EcM richness at SH – and EcM lineage-level were calculated using ANCOVA in stats 4.2.3, with sequencing depth as a covariate (Tedersoo and Smith 2013; R Core Team 2023). NMDS analysis was performed using Bray-Curtis distances on Hellinger-transformed reads at 1000 permutations and environmental variables were plotted using VEGAN 2.6.4 (Oksanen et al. 2024). Model selection and fitting for PERMANOVA was performed using AICCPERMANOVA 0.0.2 (Corcoran 2023). Indicator species were identified using presence-absence data INDICSPECIES 1.7.15 and Venn diagrams were constructed using relative abundances in MICECO 0.9.19 (Cáceres and Legendre 2009; Russel 2024). Plots were generated using GGPLOT2 3.5.1 (Wickham 2016).

To evaluate sampling and taxonomic efforts in African *Lactifluus* species, we performed SH-matching as described above on an ITS dataset of *Lactifluus* specimens collected in Africa, stored at the GENT fungarium (Belgium). We distinguished between: a) SHs that are absent from our own collection, b) those that are present and have been formally described and c) those present in the GENT fungarium, but are yet to be formally described. These collections have been assigned to species based on thorough molecular and morphological study, the majority of which have been documented in previous studies. In cases where a formal species description and publication are lacking, the collections have been assigned to species concepts using the same approach (e.g. *Lactifluus* sp. LP48). We generated a bipartite network graph to compare our taxonomy of these collections with the annotation in UNITE v.10, using the R package BIPARTITE 2.19 (Dormann et al. 2009).

## ﻿Results

### ﻿Sampling and sequencing depth

A total of 372,711 dereplicated sequences were assigned to 6712 fungal SHs at 98% similarity, after excluding low abundance SHs. Both sequencing and sampling depth, did not appear to be sufficient. Extrapolation suggests that approximately 25% additional SHs could be discovered if sequencing or sampling efforts were doubled (Suppl. material 1: fig. S1). Only 39% of the SHs could be identified at the genus level and 14% at species level. Of these SHs that were at least identified to the genus level, 345 are annotated as ectomycorrhizal (EcM), corresponding to 13% of these genus-identified SHs, 13% of total reads and 5% of all SHs.

### ﻿EcM communities and diversity across African vegetation types

**Figure 2.**
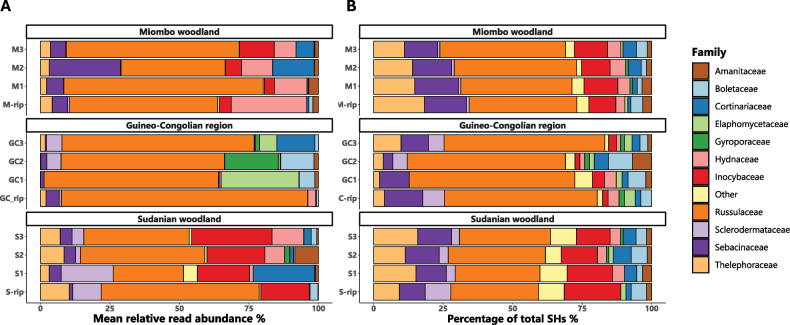
Relative abundances of ectomycorrhizal families: **A** mean relative abundance; **B** percentage of total Species Hypotheses of ectomycorrhizal families per forest subtype. Average read abundances were calculated, based on subplots per forest subtype. Riparian forests are indicated by ‘rip’. Only the 11 most abundant families are displayed: other families that contain ectomycorrhizal taxa, in decreasing abundance, are: *Pyronemataceae*, *Hymenogastraceae*, *Leotiaceae*, *Atheliaceae*, *Hydnangiaceae*, *Helvellaceae*, *Pezizaceae* and *Hymenochaetaceae*.

*Russulaceae* were by far the most dominant EcM group across all three vegetation types (Fig. 2A). *Inocybaceae*, *Sebacinaceae*, *Thelephoraceae*, *Hydnaceae* and *Cortinariaceae* were the next co-dominating EcM families in woodlands, in that order. The Guineo-Congolian rainforest was enriched in *Elaphomycetaceae*, *Boletaceae* and *Gyroporaceae*. *Amanitaceae* were relatively scarce across all forest types. Sudanian woodland was significantly richer in *Sclerodermataceae* compared to Miombo woodland (overall P = 0.045, pairwise P = 0.035) and in *Inocybaceae* and *Thelephoraceae* compared to the Guineo-Congolian Region (overall P < 0.0010, pairwise P < 0.0010 and overall P = 0.029, pairwise P = 0.021). *Sebacinaceae* and *Hydnaceae* were significantly more abundant in Miombo woodland compared to Sudanian woodland and Guineo-Congolian Region (overall P = 0.0020, pairwise P = 0.044 and 0.0020 and overall P = 0.0070, pairwise P = 0.049 and 0.0090). Lastly, *Boletaceae* were significantly richer in the rainforest compared to Miombo woodland (overall P = 0.029, pairwise P = 0.040).

The percentage of SHs per family was largely congruent with the relative abundance of that family in each subtype (Fig. 2B). *Thelephoraceae* and *Sebacinaceae* seemed to be more species-rich than expected, based on their abundances, while the opposite holds true for the *Russulaceae*, *Hydnaceae*, *Cortinariaceae* and *Elaphomycetaceae*. Within the *Elaphomycetaceae*, the Guineo-Congolian samples were almost mono-dominated by *Elaphomyceslabyrinthus* (SH0658513.10FU).

**Figure 3.**
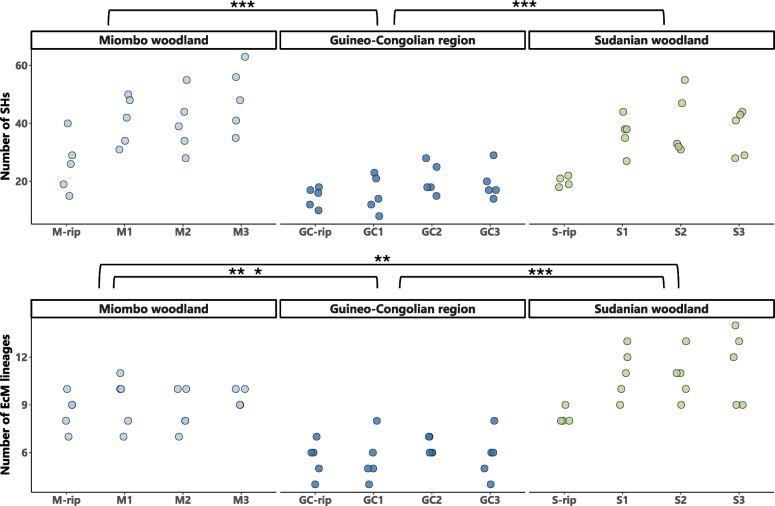
Dot plots of ectomycorrhizal diversity across different vegetation types: **A** differences in number of ectomycorrhizal SHs; **B**EcM lineages are illustrated between Miombo woodland, Guineo-Congolian region and Sudanian woodland, as well as between their respective subtypes (*P < 0.05, **P < 0.01, ***P < 0.001).

The diversity of EcM fungi at SH level significantly differed between the Guineo-Congolian Region and Miombo woodland and between the Guineo-Congolian Region and Sudanian woodland (overall P = 1e^-05^, pairwise P < 1e^-04^), with the rainforest consistently showing lower species richness (Fig. 3). Riparian plots within Miombo and Sudanian woodlands also exhibited lower diversity, yet only a single sample pair (M3-M_rip) yielded a significant difference (pairwise P < 0.01). At the level of EcM lineages, differences between subtypes were less pronounced. Between forest types, however, significant differences in richness could be detected (overall P = 3.87e^-12^). Sudanian woodland displayed a higher richness than Miombo (pairwise P = 0.0050) and the Guineo-Congolian Region (pairwise P < 1e^-04^), while Miombo woodland exhibited higher richness than the Guineo-Congolian region (pairwise P < 1e^-04^). Sequencing depth was included as a covariate in these analyses, but yielded no significant effect.

**Figure 4.**
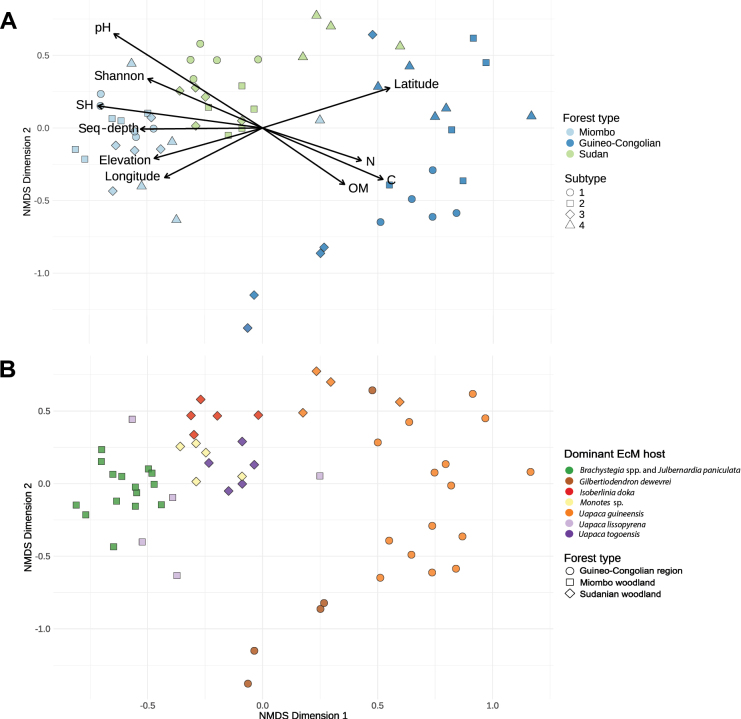
NMDS of Bray-Curtis distances of ectomycorrhizal Hellinger-transformed Species Hypotheses: **A** significant variables are plotted and samples are coloured according to forest type (Miombo woodland, Guineo-Congolian Region and Sudanian woodland) and are shaped according to forest subtype, with triangles as riparian forests; **B** samples are coloured according to dominant ectomycorrhizal host in these forest subtypes and are shaped according to forest type.

The composition of ectomycorrhizal (EcM) fungal communities differed greatly between the three vegetation types, as visualised in an NMDS with stress = 0.2027 (Fig. 4). PERMANOVA results indicate that a model with only forest type explained 15% of the variation in community composition when correcting for sequencing depth and a model with only forest subtype explained 41% (Suppl. material 1: table S1). These differences between the vegetation types are linked to soil characteristics or edaphic factors, which vary significantly across our considered ecosystems with their respective distinct soil profiles (Fig. 4A). In Miombo and Sudanian woodlands, soils tend to have a neutral to slightly alkaline pH and are generally nutrient poor, with low levels of carbon (C), nitrogen (N) and organic material. In contrast, rainforest soils often have low pH values and are rich in organic material, carbon and nitrogen, providing a more nutrient-dense substrate. These edaphic factors are, however, difficult to disentangle from geography, as latitude, longitude and elevation were also significantly correlated with the ordination axes of the NMDS (Fig. 4A).

However, dominant EcM host in each vegetation subtype also seemed to explain a large degree of variation in the dataset. A model with only dominant EcM host, accounting for sequencing depth, produced a lower AICc than the previously discussed models and, here, the dominant host explains 28% of the variation (Suppl. material 1: table S1; Fig. 4B).

**Figure 5.**
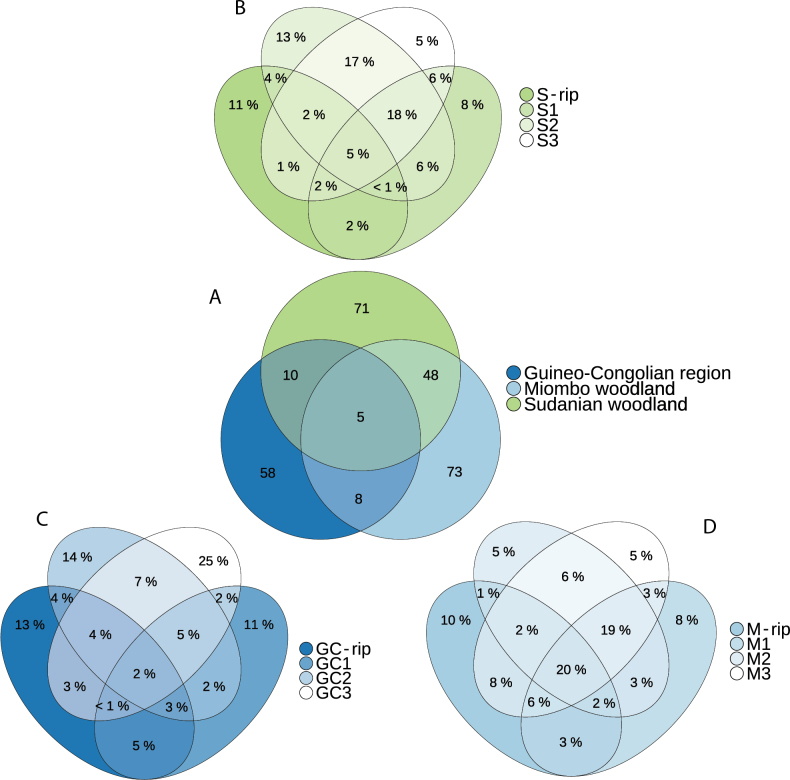
Venn diagram of ectomycorrhizal Species Hypotheses shared between vegetation types and subtypes: **A** number of unique Species Hypotheses are displayed for Miombo woodland, Sudanian woodland and Guineo-Congolian Region, with overlapping regions illustrating shared diversity; **B–D** relative proportions of Species Hypotheses shared between subtypes of Sudanian woodland, Guineo-Congolian Region and Miombo woodland, respectively.

Despite the differences observed in the NMDS and the large geographical distance between both, Sudanian and Miombo woodlands shared a substantial number (40%) of SHs (Fig. 5A). The Guineo-Congolian Region only shared 19% and 16% of its SHs with the Sudanian and Miombo woodlands, respectively. Five SHs were found across all vegetation types. To understand how these SHs are distributed across the different subtypes within the three vegetation types, the relative distribution of SHs between subtypes was analysed (Fig. 5B–D). A total of 63% of taxa in the Guineo-Congolian Region were unique to the sampling locality, whilst this is only 37% for Sudanian woodland and 38% for Miombo, illustrating a lower degree of shared fungal community between rainforest plots. Moreover, Miombo woodland displayed a large degree of generalist taxa: 20% were shared amongst all subtypes. Lastly, indicator SHs for specific forest types or subtypes are listed in Suppl. material 3: table S2.

### ﻿Taxonomic completeness in African *Lactifluus*

**Figure 6.**
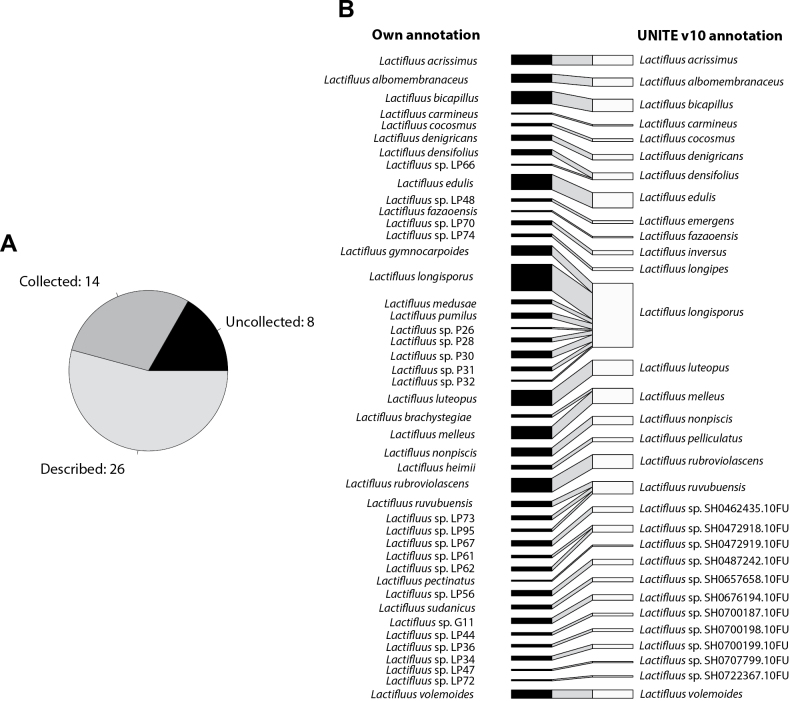
Comparison of *Lactifluus* collections in GENT fungarium and *Lactifluus* Species Hypotheses identified in soil samples: **A** distribution of Species Hypothesis annotation at the species level in African *Lactifluus*. Uncollected: no annotation at the species level in UNITE nor present in the GENT fungarium, described: has an associated species epithet in our collection and collected: present in collection at the GENT fungarium awaiting formal description; **B** bipartite network graph comparing taxonomic annotation of: i) our own collections (left) and ii) their respective Species Hypotheses (2%) in UNITE (right). Only those Species Hypotheses that were matched in the metabarcoding dataset were used for constructing this plot.

The African *Lactifluus* species found in this dataset are well-documented (Fig. 6A). Approximately half of the SHs (26) have been collected and are represented in our fungarium with annotation of a valid species name and are considered here as ‘described’. Another third (14) additional SHs have been collected during previous expeditions and are preserved in the GENT fungarium, but have not yet been formally described (‘collected’). Only eight of the 48 SHs identified in the metabarcoding dataset (17%) lacked representation in our fungarium collection (‘uncollected’) and could be considered ‘dark taxa’ that cannot be linked to a physical specimen (Ryberg and Nilsson 2018). The SHs that remain undescribed and uncollected were evenly distributed across vegetation types, with three SHs occurring in the Miombo woodland, two in the Sudanian woodland and three in the Guineo-Congolian Region.

To evaluate how the species names annotated in our collection align with UNITE annotations and to determine the effectiveness of SH matching in reflecting our species concepts, we constructed a bipartite network graph (Fig. 6B). This graph provides insights into the correspondence between our curated taxonomy and the molecular species hypotheses from metabarcoding data.

## ﻿Discussion

### ﻿Diversity of below-ground EcM communities across vegetation types

The diversity observed in root-tip analyses of underground ectomycorrhizal (EcM) fungal communities partially aligns with findings from fruit-body-based research. Consistent with fruit-body inventories, *Russulaceae* dominate EcM fungal communities across all vegetation types, with *Lactifluus* and *Russula* being the most abundant genera (Verbeken and Buyck 2002). *Sclerodermataceae* read counts are lower compared to *Russulaceae*, but similarly reflect their above-ground abundance. In contrast, *Boletaceae*, *Amanitaceae* and *Cantharellus* spp. (*Hydnaceae*) are under-represented below-ground. *Boletaceae* are better represented in some Guineo-Congolian plots, but are especially sparse in woodland areas, except for a slightly higher abundance in riparian woodland. This distribution contrasts with the high abundance of bolete fruit-bodies in woodland environments. Similar trends, including enrichment of *Boletaceae* in riparian plots, have been reported previously in west-African woodlands (Meidl et al. 2021). The apparent discrepancy may result from high seasonal mycelial turnover (Badou et al. 2022) or limited sampling of different soil layers, although previous studies suggest low soil stratification and associated EcM niche differentiation in African woodlands (Tedersoo et al. 2011). More likely, the long ITS1 and ITS2 regions of *Boletaceae* results in bias during PCR and short-read sequencing. Conversely, the low abundances of *Hydnaceae*, which include the chanterelles, could be attributed to primer mismatching, as certain taxa are more prone to mismatch because of their unique or divergent sequences within targeted regions (Tedersoo et al. 2015).

Opposite patterns can be observed in other clades, such as *Thelephoraceae*, which show a high below-ground abundance compared to their relatively low above-ground records, which is undoubtedly related to their inconspicuous fruit-body formation, as has been observed in other studies (Tedersoo et al. 2014b; Meidl et al. 2021). Additionally, *Inocybaceae* and *Cortinariaceae* show surprisingly high read abundances, especially in Sudanian woodland. Likewise, the read abundances of *Sebacinaceae* are surprisingly high. Ecological guilds within the *Sebacinaceae* are diverse, encompassing species functioning as EcM symbionts, orchid mycorrhizal partners and even endophytes (Tedersoo et al. 2014a). However, all SHs in our dataset are mapped to the genus *Sebacina*, which contains only EcM (Selosse et al. 2002; Tedersoo and Smith 2017). The discrepancy between below – and above-ground abundances underscores the importance of integrating multiple datasets to achieve a more comprehensive understanding of fungal biodiversity.

### ﻿EcM community variation between forest types and subtypes

The EcM fungal communities differ significantly between forest types and even more when discriminating between riparian and non-riparian plots. These differences seem to be linked to edaphic factors, geographical location and dominant EcM host. Guineo-Congolian EcM communities are especially different from woodlands, despite their geographical position in-between the Sudanian and Miombo woodlands. The degree of shared diversity between different rainforest subtypes is almost half of those between the woodland’s subtypes, which could be explained by the isolation of different rainforest plots within AMF-dominated stands. This confirms the hypothesis that the spatial structure of isolated EcM stands promotes rare and potentially endemic EcM communities in rainforests of the Guineo-Congolian Region.

Distinctions between non-riparian subtypes, particularly within Miombo woodland, are less pronounced than between riparian and non-riparian subplots. This likely results from the low variability between subtypes compared to the other vegetation types, as illustrated by the consistent co-dominating mixture of EcM hosts and lower geographical distance between sampling locations within Miombo woodland plots (Fig. 1). The same pattern can be deduced from the large degree of shared fungal taxa amongst all subtypes in Miombo woodlands: 20% compared to 5% and 2% in Sudanian woodland and the Guineo-Congolian Region (Fig. 5B–D). In contrast, Guineo-Congolian rainforest and Sudanian woodland plots show greater variation between subtypes due to differences in dominant EcM hosts and larger geographical spacing between plots. For instance, in the rainforest, the subtype dominated by *Gilbertiodendrondewevrei* (RF3) differs markedly from those dominated by *Uapacaguineensis*, despite their geographic proximity. These results highlight the importance of host specificity in shaping EcM fungal communities, contrasting with earlier studies suggesting low host specificity within Central African rainforests and woodlands compared to temperate regions (Verbeken and Buyck 2002; Tedersoo et al. 2011; Meidl et al. 2021). While shared EcM communities may dominate at local scales within rainforest plots (Diédhiou et al. 2010), our findings thus suggest that dominant hosts strongly influence fungal community composition at a larger scale. However, at this scale, it is difficult to disentangle these patterns from correlated edaphic factors. For example, the rainforest plots dominated by *Gilbertiodendron* display a lower level of humidity, which also influences the EcM community.

Miombo and Sudanian woodland vegetation exhibit significant overlap in ectomycorrhizal (EcM) fungal diversity, despite their large geographical separation (Fig. 1). This pattern is also supported by the indicator species analysis. For instance, *Lactifluusluteopus* (SH0677010.10FU) (*Russulaceae*) is a common species found across both woodland types and their riparian areas (Verbeken 1995). Other species, such as *Lactifluusvolemoides* (SH0558869.10FU) (*Russulaceae*) and *Tomentellabrunneocystidia* (SH0618277.10FU) (*Thelephoraceae*), are restricted to the dry woodland areas from which they were originally described (Karhula et al. 1998; Yorou et al. 2007). Indicator species analyses further reveal the presence of some *Cortinarius* spp. (SH0675158.10FU, SH0702270.10FU) and *Inocybe* spp. (SH0540475.10FU, SH0612229.10FU) as prominent in these (riparian) woodlands. These species were mistakenly identified as European species, likely due to the limitations of relying solely on the ITS2 region and the application of a conservative similarity threshold and may represent undescribed species (Garnica et al. 2016). This aligns with the broader context of African fungal diversity, where a significant amount of species remains unexplored (Piepenbring et al. 2020). These findings highlight the challenges of accurate species identification within understudied fungal taxa and regions, particularly in the complex and biodiverse tropical ecosystems and underscore the need for continued taxonomic research to uncover and document the true extent of ectomycorrhizal (EcM) fungal diversity in African ecosystems. Such efforts are essential in advancing our understanding their ecological roles in these unique and understudied regions.

### ﻿Riparian forests as a corridor for rare taxa

Riparian forests within Sudanian woodlands share a strong resemblance in ectomycorrhizal (EcM) community composition with riparian areas of the Guineo-Congolian Region. In contrast, Miombo riparian forests show less similarity to the riparian Guineo-Congolian plots, with only one Miombo riparian subplot exhibiting high resemblance. This difference may reflect the greater geographical distance between Miombo and Guineo-Congolian plots compared to Sudanian and Guineo-Congolian plots. Additionally, Sudanian riparian and Guineo-Congolian riparian forests share the same dominant EcM host, *Uapacaguineensis*, which may further explain their community overlap. Moreover, Guineo-Congolian subtypes 1 and 2 also share the same host and show a high similarity in EcM community to these riparian plots, further emphasising the importance of dominant EcM host.

As hypothesised from field observations, the humid conditions in riparian forests and rainforests foster a distinct EcM fungal composition. These habitats are enriched in *Boletaceae*, *Gyroporaceae* and *Elaphomycetaceae*, but exhibit lower EcM diversity compared to woodlands. Indicator species such as *Russulabrunneoannulata* (SH0487246.10FU) and *Afroboletusluteolus* (SH0640896.10FU) (*Boletaceae*) are characteristic of Miombo riparian forests, while *Lactifluusmelleus* (SH0658331.10FU) and *Lf.fazaoensis* (SH0559923.10FU) are prominent in Sudanian riparian forests. Guineo-Congolian riparian forests feature unique taxa such as *Lf.albomembranaceus* (SH0612414.10FU) and *Lf.rubroviolascens* (SH0472911.10FU), while the rainforest habitats host rare species like *Elaphomyceslabyrinthinus* (SH0658513.10FU) and *Komboclesbakaiana* (SH0568085.10FU) (*Boletaceae*). *K.bakaiana*, a rare sequestrate bolete, exemplifies the potential for endemicity in these ecosystems. It is known only from the rainforest sampling site, its type locality (Castellano et al. 2016). Similarly, *Lf.albomembranaceus* has only been documented in the Guineo-Congolian riparian locality, further underscoring the distinct and unique nature of this region’s EcM fungal community (De Crop et al. 2016). On the other hand, species such as *Russulapseudocarmenisa* (SH0573140.10FU), found in both Guineo-Congolian and Sudanian riparian forests, demonstrate the role of riparian areas as ecological corridors, allowing rainforest-adapted species to establish within these distinct vegetation types.

To summarise, at a large spatial scale, the EcM community composition is primarily influenced by vegetation type and correlated edaphic factors. However, disentangling these drivers from distance decay is challenging due to spatial autocorrelation (Corrales et al. 2018). At finer, local scales within vegetation types, the dominant EcM host tree seems to exert strong effects on the EcM fungal community composition. At an even lower scale, within plots, host-specificity is likely less pronounced. Yet, these host-driven effects are modulated by, and difficult to separate from, the differing local edaphic and climatic conditions, particularly the similar humid environments of riparian forests and rainforests. Riparian forests may act as ecological corridors for rainforest-associated species, particularly when the spatial separation between habitats is moderate. This highlights their critical role in maintaining connectivity and facilitating species exchange across distinct ecological zones.

### ﻿Taxonomic framework and accuracy of SH-matching in African *Lactifluus*

Our results confirm that *Lactifluus* is one of the best-studied ectomycorrhizal (EcM) genera in Africa, with nearly 75% of the identified Species Hypotheses (SHs) represented as collections in the GENT fungarium. This extensive coverage provides a robust basis for evaluating both taxonomic completeness (i.e. the proportion of collected and/or described species) and the accuracy of SH matching using a fixed threshold compared to detailed taxonomic study.

Overall, the identified SHs correspond well to their respective fungarium identifications when using the 2% threshold. Mismatches between ITS-based SH-matching and our own taxonomic assignments, based on morphology and more elaborate sequence data, mostly involve species complexes, such as *Lactifluuslongisporus* s.l. and *Lf.ruvubuensis* s.l. (Verbeken 1998; Verbeken et al. 2012; Maba et al. 2014; De Crop et al. 2017). For instance, the *Lf.longisporus* s.l. species complex represents a particularly interesting case, as it includes some of the most abundant edible EcM species in African woodlands, such as *Lf.gymnocarpoides* and *Lf.longisporus* s.s. (De Kesel et al. 2024). However, at a 2% threshold in UNITE, all sequences from this complex are grouped under SH0677016.10FU, which includes the type specimen of *Lf.longisporus*. A failure to recognise all taxa within a species complex, as illustrated here when employing a fixed clustering threshold, leads to underestimation and poor recognition of both ecologically and ethnobiologically/economically important taxa. However, datasets generated at different similarity or clustering thresholds have been shown to yield highly similar results in large-scale ecological analyses (Botnen et al. 2018).

Unclassified SHs that lack fungarium representation are often based solely on environmental sequences originating from roots or soil, many of which are represented by singletons. These SHs likely correspond to rare species, taxa that infrequently form fruit-bodies or represent sequencing artefacts. In some cases, collections without a valid species name are linked to an SH with a valid name (e.g. *Lf.emergens*, SH0707802.10FU). Vice versa, some collections that have a taxonomic annotation in our collections, such as *Lf.sudanicus*, are linked to an SH without a species annotation. In either case, there is no sequence available of a type specimen, resulting either in a wrong taxonomic annotation in UNITE or no taxonomic annotation in UNITE, but was the name given to our collection by choosing our own, unofficial, reference sequence. The only true taxonomic mismatch unrelated to similarity thresholds is between *Lf.heimii* and *Lf.pelliculatus*. Despite the inclusion of the holotype of *Lf.heimii* in SH0700212.10FU, it has not been chosen as the reference sequence, resulting in an incorrect annotation in the UNITE database. A correction has been submitted for a taxonomic re-annotation of this SH cluster.

### ﻿Methodological considerations

This study allows us to conclude with some considerations regarding the sampling strategy. Our approach of assessing eDNA from roots rather than soil samples proved to be both labour-intensive and time-consuming. The goal was to capture a more complete ectomycorrhizal (EcM) fungal community associated with a specific host plant. However, given that only 13% of the genus-level identifiable SHs consists of ectomycorrhizal representatives, we question the added value of this method compared to soil sampling. As it proved impossible to completely remove soil particles from the roots, many of these non-EcM taxa likely originate from these residual particles. Likewise, it is possible that not all EcM identified here are truly colonising the tree roots. To better understand the relative merits of these approaches, more direct comparative research is needed, involving eDNA analyses from root tips and soil samples collected from the same plots. Additionally, we recognise limitations in the selection of our study sites. For example, the lower richness in EcM-lineages in Miombo woodland compared to Sudanian woodland can likely be attributed to the smaller covered sampling area in Miombo, rather than a true biological phenomenon. We do, however, believe that the Guineo-Congolian Region is less diverse compared to the woodlands, both in species richness as in EcM-lineages, as the sampling area covered is larger than the Miombo and Sudanian woodland. Ideally, the distances between different plots in every vegetation type would be standardised to ensure comparability. However, this was not feasible due to the logistical challenges of working in Africa, where site selection depends heavily on accessible areas, the availability of local partners and other practical constraints.

## ﻿Conclusions

In this study, we characterise the ectomycorrhizal (EcM) community across three African vegetation types using root-tip metabarcoding: Sudanian woodland, Miombo woodland and Guineo-Congolian forests. Each vegetation type shows a distinct EcM community, reflecting each of their ecology. Notably, riparian forests within the Sudanian woodlands and Guineo-Congolian zone show a high resemblance to the EcM communities of Guineo-Congolian rainforests. These riparian forests may act as ecological corridors, facilitating the migration of rare and specialised rainforest-associated EcM taxa into adjacent woodland areas. Thus, local edaphic conditions can override distance decay in shaping the community structure of African EcM. We also show that host specificity shapes EcM community composition at a large spatial scale, in contrast to previous studies.

Across all three vegetation types, *Russulaceae* is confirmed as the dominant EcM clade, consistent with its known prevalence in African ecosystems. However, there are pronounced differences in the abundances of other EcM clades between below – and above-ground diversity. The accuracy of the SH-matching tool was evaluated by comparing metabarcoding results with our fungarium collections of the genus Lactifluus (Russulaceae). Over 80% of the identified SHs correspond to specimens in our herbarium, highlighting the utility of SH-matching for taxonomic classification in genera with good reference data. The SHs generated through the SH matching tool align well with our own species concepts based on our collections. However, the approach shows limitations, particularly in resolving species complexes.

## ﻿Additional information

### Adherence to national and international regulations

The collection and use of the samples and genetic resources are in compliance with the legal and ethical frameworks established by the respective nations of origin at the time of acquisition (Cameroon MINRESI/B00/C00/C10/C12, Togo 105206/14/DPV, Zambia 17/02/2014 University of Zambia).

### Author ORCIDs

Lowie Tondeleir https://orcid.org/0009-0008-5951-1709

Eske De Crop https://orcid.org/0000-0002-9067-6981

Jorinde Nuytinck https://orcid.org/0000-0001-9250-377X

André-Ledoux Njouonkou https://orcid.org/0000-0002-8375-024X

Glen Dierickx https://orcid.org/0000-0001-5387-8544

József Geml https://orcid.org/0000-0001-8745-0423

Annemieke Verbeken https://orcid.org/0000-0002-6266-3091

### Data availability

Raw sequence data have been submitted to GenBank SRA under BioProject number PRJNA1228630. ITS sequences for the comparative analysis of *Lactifluus* were submitted to GenBank under accession numbers PV135576 to PV135705. The SH table used for the statistical analyses, as well as a table containing the metadata for each sample, are provided as supplementary data.

## Supplementary materials

Supplementary material 1 Supplementary figure and table Data type doc Explanation note **figure S1**: Sequencing and sampling depth across the vegetation types; **table S1**: PERMANOVA results of Bray-Curtis distances on Hellinger-transformed data. **table S2**: Ectomycorrhizal indicator species for each forest subtype. https://binary.pensoft.net/file/1341164This dataset is made available under the Open Database License (http://opendatacommons.org/licenses/odbl/1.0/). The Open Database License (ODbL) is a license agreement intended to allow users to freely share, modify, and use this Dataset while maintaining this same freedom for others, provided that the original source and author(s) are credited.Lowie Tondeleir

Supplementary material 2 Metadata of all samples Data type xlsx Explanation note Sample ID, pH, C, N, P, OM, elevation, coordinates, country, plot information and dominant EcM hosts. https://binary.pensoft.net/file/1341165This dataset is made available under the Open Database License (http://opendatacommons.org/licenses/odbl/1.0/). The Open Database License (ODbL) is a license agreement intended to allow users to freely share, modify, and use this Dataset while maintaining this same freedom for others, provided that the original source and author(s) are credited.Lowie Tondeleir

Supplementary material 3 SH table Data type csv Explanation note table with read abundances of Species Hypotheses (SHs) per sample. https://binary.pensoft.net/file/1341166This dataset is made available under the Open Database License (http://opendatacommons.org/licenses/odbl/1.0/). The Open Database License (ODbL) is a license agreement intended to allow users to freely share, modify, and use this Dataset while maintaining this same freedom for others, provided that the original source and author(s) are credited.Lowie Tondeleir
