# Supplementary material for: ﻿Back to the roots: Uncovering ectomycorrhizal communities across three major African vegetation types
Source: IMA Fungus. 2025 May 29;16:e147055. doi: 10.3897/imafungus.16.147055 (PMC12142211; doi:10.3897/imafungus.16.147055)
Supplement: Supplementary material 1 — Supplementary figure and table [file imafungus-16-e147055-s001.doc]

**Suppl. material 1**

# Supplementary figure and tables


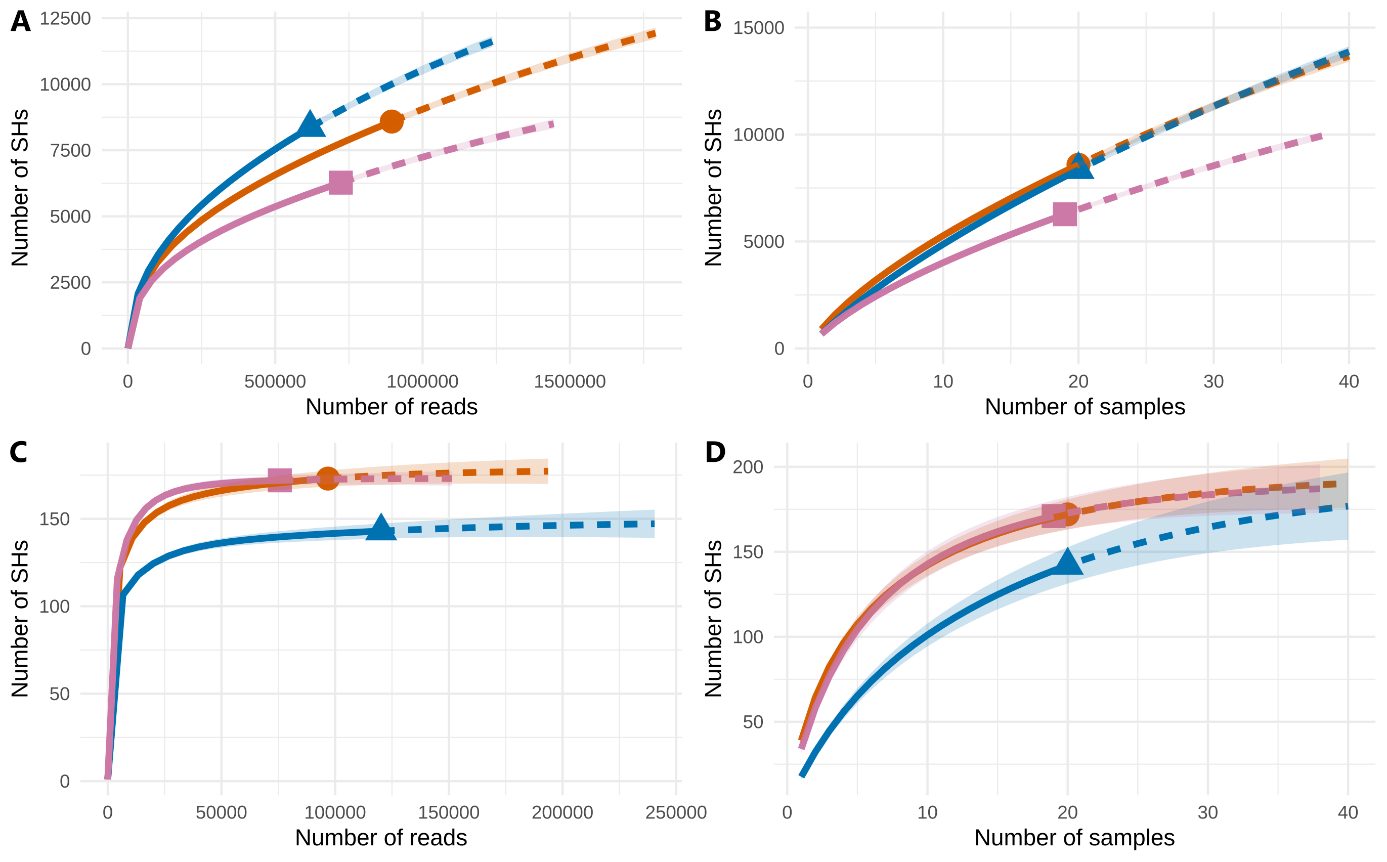


**Supplementary Figure S1.** Sequencing and sampling depth across the vegetation types. **A** Number of total fungal Species Hypotheses (before removing low-abundances) are displayed relative to sequencing depth; **B** sampling depth. Full lines indicate rarefaction curves, interrupted lines indicate extrapolation. Orange/circle: Miombo woodland, blue/triangle: Guineo-Congolian Region, pink/square: Sudanian woodland.

**Supplementary Table S1. PERMANOVA results of Bray-Curtis distances on Hellinger-transformed data. Variance inflation factor (VIF), AIC criterion (AICc), number of parameters (k) and adjusted R^2^ for each parameter are given for each model. Models with VIF > 20 were excluded.**

| **Model** | **VIF** | **AICc** | **k** | **Forest type** | **Forest subtype** | **Sequencing depth** | **Dominant EcM host** |
| --- | --- | --- | --- | --- | --- | --- | --- |
| Distance ~ Dominant EcM host | 0 | -58,47 | 7 | NA | NA | NA | 0,3181 |
| Distance ~ Forest type | 0 | -57,99 | 3 | 0,1889 | NA | NA | NA |
| Distance ~ Sequencing depth + Dominant EcM host | 6 | -57,42 | 8 | NA | NA | 0,0186 | 0,2775 |
| Distance ~ Forest type + Sequencing depth | 2 | -57,29 | 4 | 0,1515 | NA | 0,0218 | NA |
| Distance ~ Forest subtype | 0 | -56,34 | 12 | NA | 0,4479 | NA | NA |
| Distance ~ Forest subtype + Sequencing depth | 11 | -54,81 | 13 | NA | 0,4051 | 0,0164 | NA |
| Distance ~ Sequencing depth | 0 | -51,46 | 2 | NA | NA | 0,0593 | NA |

**Supplementary Table S2. Ectomycorrhizal indicator species for each forest subtype. Indicator values (stat > 0.50) and P (> 0.025) of species level-identified Species Hypotheses are displayed.**

| **SH** | **stat** | **P** | **Taxonomy UNITE v.10.0** |
| --- | --- | --- | --- |
| **Miombo** |  |  |  |
| SH0529980.10FU | 0.960 | 0.001 | *Russula* sp. |
| SH0586121.10FU | 0.791 | 0.001 | *Russula* sp. |
| SH0588818.10FU | 0.745 | 0.001 | *Russula* sp. |
| SH0676733.10FU | 0.745 | 0.001 | *Russula ochraceorivulosa* |
| SH0662043.10FU | 0.740 | 0.001 | *Inocybe* sp. |
| SH0707799.10FU | 0.698 | 0.001 | *Lactifluus* sp. |
| SH0631282.10FU | 0.670 | 0.001 | *Sebacina* sp. |
| SH0700198.10FU | 0.614 | 0.003 | *Lactifluus* sp. |
| SH0591249.10FU | 0.598 | 0.014 | *Lactifluus* sp. |
| SH0469165.10FU | 0.561 | 0.010 | *Sebacina* sp. |
| SH0472926.10FU | 0.542 | 0.013 | *Lactifluus edulis* |
| SH0594782.10FU | 0.542 | 0.015 | *Sebacina* sp. |
| SH0621125.10FU | 0.542 | 0.012 | *Tomentella* sp. |
| SH0697218.10FU | 0.542 | 0.011 | *Hydnobolites* sp. |
|  |  |  |  |
| **Miombo-riparian** |  |  |  |
| SH0570118.10FU | 0.652 | 0.008 | *Sebacina* sp. |
| SH0631110.10FU | 0.614 | 0.015 | *Sebacina* sp. |
| SH0640896.10FU | 0.614 | 0.012 | *Afroboletus luteolus* |
| SH0487246.10FU | 0.598 | 0.015 | *Russula brunneoannulata* |
|  |  |  |  |
| **Guineo-Congolian** |  |  |  |
| SH0675914.10FU | 0.598 | 0.014 | *Tylopilus* sp. |
| SH0658513.10FU | 0.546 | 0.010 | *Elaphomyces labyrinthinus* |
| SH0463720.10FU | 0.542 | 0.008 | *Russula* sp. |
| SH0529982.10FU | 0.542 | 0.011 | *Russula* sp. |
| SH0573165.10FU | 0.542 | 0.014 | *Russula* sp. |
| SH0640897.10FU | 0.542 | 0.009 | *Afroboletus* sp. |
| SH0568085.10FU | 0.538 | 0.022 | *Kombocles bakaiana* |
|  |  |  |  |
| **Guineo-Congolian-riparian** |  |  |  |
| SH0472911.10FU | 0.760 | 0.001 | *Lactifluus rubroviolascens* |
| SH0650830.10FU | 0.652 | 0.007 | *Clavulina* sp. |
| SH0551450.10FU | 0.598 | 0.017 | *Tomentella* sp. |
| SH0612414.10FU | 0.598 | 0.017 | *Lactifluus albomembranaceus* |
|  |  |  |  |
| **Sudanian** |  |  |  |
| SH0488778.10FU | 0.811 | 0.001 | *Wilcoxina rehmii* |
| SH0540475.10FU | 0.745 | 0.001 | *Inocybe catalaunica* |
| SH0675158.10FU | 0.745 | 0.001 | *Cortinarius subpaleaceus* |
| SH0609048.10FU | 0.718 | 0.001 | *Scleroderma* sp. |
| SH0662359.10FU | 0.698 | 0.001 | *Inocybe* sp. |
| SH0692394.10FU | 0.649 | 0.002 | *Inocybe* sp. |
| SH0702270.10FU | 0.649 | 0.003 | *Cortinarius laetus* |
| SH0460146.10FU | 0.598 | 0.009 | *Sebacina* sp. |
| SH0588831.10FU | 0.598 | 0.014 | *Russula* sp. |
| SH0616942.10FU | 0.598 | 0.014 | *Tomentella* sp. |
| SH0653912.10FU | 0.598 | 0.013 | *Lactarius controversus* |
| SH0650742.10FU | 0.561 | 0.010 | *Russula* sp. |
| SH0552508.10FU | 0.542 | 0.012 | *Amanita* sp. |
| SH0619949.10FU | 0.542 | 0.017 | *Tomentella* sp. |
|  |  |  |  |
| **Sudanian-riparian** |  |  |  |
| SH0612229.10FU | 1.000 | 0.001 | *Inocybe pallidiangulata* |
| SH0460148.10FU | 0.845 | 0.001 | *Sebacina* sp. |
| SH0609034.10FU | 0.845 | 0.001 | *Scleroderma* sp. |
| SH0658331.10FU | 0.845 | 0.001 | *Lactifluus melleus* |
| SH0657658.10FU | 0.801 | 0.002 | *Lactifluus* sp. |
| SH0644275.10FU | 0.742 | 0.001 | *Russula* sp. |
| SH0625353.10FU | 0.674 | 0.005 | *Inocybe* sp. |
| SH0675895.10FU | 0.634 | 0.010 | *Scleroderma* sp. |
| SH0559923.10FU | 0.620 | 0.004 | *Lactifluus fazaoensis* |
| SH0632846.10FU | 0.574 | 0.019 | *Scleroderma* sp. |
|  |  |  |  |
| **Miombo + Miombo-riparian** |  |  |  |
| SH0620076.10FU | 0.877 | 0.001 | *Tomentella* sp. |
| SH0680820.10FU | 0.780 | 0.001 | *Amanita* sp. |
| SH0480123.10FU | 0.658 | 0.002 | *Russula* sp. |
| SH0631082.10FU | 0.641 | 0.001 | *Sebacina* sp. |
| SH0587348.10FU | 0.607 | 0.010 | *Russula brunneoderma* |
| SH0464374.10FU | 0.596 | 0.012 | *Pulveroboletus* sp. |
| SH0480117.10FU | 0.581 | 0.016 | *Russula* sp. |
| SH0573139.10FU | 0.528 | 0.017 | *Russula* sp. |
| SH0631215.10FU | 0.500 | 0.018 | *Sebacina* sp. |
|  |  |  |  |
| **Miombo + Guineo-Congolian** |  |  |  |
| SH0573173.10FU | 0.581 | 0.003 | *Russula hiemisilvae* |
|  |  |  |  |
| **Miombo + Sudanian** |  |  |  |
| SH0460150.10FU | 0.732 | 0.001 | *Sebacina* sp. |
| SH0631347.10FU | 0.707 | 0.001 | *Sebacina* sp. |
| SH0650837.10FU | 0.693 | 0.001 | *Clavulina* sp. |
| SH0644285.10FU | 0.682 | 0.001 | *Lactarius* sp. |
| SH0619005.10FU | 0.640 | 0.003 | *Tomentella* sp. |
| SH0588809.10FU | 0.632 | 0.001 | *Russula* sp. |
| SH0565687.10FU | 0.586 | 0.009 | *Tomentella* sp. |
| SH0588839.10FU | 0.555 | 0.009 | *Russula* sp. |
| SH0618227.10FU | 0.529 | 0.014 | *Tomentella brunneocystidia* |
| SH0558869.10FU | 0.500 | 0.015 | *Lactifluus volemoides* |
|  |  |  |  |
| **Guineo-Congolian-riparian + Sudanian-riparian** |  |  |  |
| SH0573138.10FU | 0.594 | 0.004 | *Russula* sp. |
| SH0573140.10FU | 0.594 | 0.004 | *Russula pseudocarmesina* |
|  |  |  |  |
| **Sudanian + Sudanian-riparian** |  |  |  |
| SH0682270.10FU | 0.583 | 0.005 | *Hebeloma mesophaeum* |
|  |  |  |  |
| **Miombo + Miombo-riparian + Sudanian + Sudanian-riparian** |  |  |  |
| SH0677010.10FU | 0.598 | 0.004 | *Lactifluus luteopus* |
